# Positive Regulatory Control Loop between Gut Leptin and Intestinal GLUT2/GLUT5 Transporters Links to Hepatic Metabolic Functions in Rodents

**DOI:** 10.1371/journal.pone.0007935

**Published:** 2009-11-30

**Authors:** Yassine Sakar, Corinne Nazaret, Philippe Lettéron, Amal Ait Omar, Mathilde Avenati, Benoît Viollet, Robert Ducroc, André Bado

**Affiliations:** 1 INSERM, U773, Centre de Recherche Biomédicale Bichat Beaujon, UFR de Médecine Paris 7 - Denis Diderot, IFR02 Claude Bernard, Paris, France; 2 Institut Cochin, Université Paris Descartes, CNRS (UMR 8104), Paris, France; 3 INSERM, U567, Paris, France; University of Las Palmas de Gran Canaria, Spain

## Abstract

**Background and Aims:**

The small intestine is the major site of absorption of dietary sugars. The rate at which they enter and exit the intestine has a major effect on blood glucose homeostasis. In this study, we determine the effects of luminal leptin on activity/expression of GLUT2 and GLUT5 transporters in response to sugars intake and analyse their physiological consequences.

**Methodology:**

Wistar rats, wild type and AMPKα_2_^−/−^ mice were used. *In vitro* and *in vivo* isolated jejunal loops were used to quantify transport of fructose and galactose in the absence and the presence of leptin. The effects of fructose and galactose on gastric leptin release were determined. The effects of leptin given orally without or with fructose were determined on the expression of GLUT2/5, on some gluconeogenesis and lipogenic enzymes in the intestine and the liver.

**Principal Findings:**

First, *in vitro* luminal leptin activating its receptors coupled to PKCβII and AMPKα, increased insertion of GLUT2/5 into the brush-border membrane leading to enhanced galactose and fructose transport. Second *in vivo*, oral fructose but not galactose induced in mice a rapid and potent release of gastric leptin in gastric juice without significant changes in plasma leptin levels. Moreover, leptin given orally at a dose reproducing comparable levels to those induced by fructose, stimulated GLUT5-fructose transport, and potentiated fructose-induced: *i)* increase in blood glucose and mRNA levels of key gluconeogenesis enzymes; *ii)* increase in blood triglycerides and reduction of mRNA levels of intestinal and hepatic Fasting-induced adipocyte factor (Fiaf) and *iii)* increase in SREBP-1c, ACC-1, FAS mRNA levels and dephosphorylation/activation of ACC-1 in liver.

**Conclusion/Significance:**

These data identify for the first time a positive regulatory control loop between gut leptin and fructose in which fructose triggers release of gastric leptin which, in turn, up-regulates GLUT5 and concurrently modulates metabolic functions in the liver. This loop appears to be a new mechanism (possibly pathogenic) by which fructose consumption rapidly becomes highly lipogenic and deleterious.

## Introduction

The small intestine is involved in delivering sugars to the systemic circulation through absorption of the products arising from carbohydrate digestion. The rate at which dietary sugars enter and exit the intestinal epithelium has a major effect on blood glucose concentration and homeostasis. Briefly, dietary carbohydrates are taken into the enterocytes by specific transporters and exit the cell through the basolateral GLUT2 transporter [1], [2]. In pre-prandial state, glucose transport is an active process which involves the co-transport of sugar with sodium ions through the sodium-glucose transporter-1, SGLT-1. In prandial state, when higher concentrations of glucose or galactose are found in the intestinal lumen, apical GLUT2 becomes active providing the small intestine with an absorptive capacity to match dietary intake during meal [1]. This apical GLUT2 transporter also participates to fructose transport in addition to the main and specific GLUT5 transporter [3]. All these membrane transporters are highly regulated during food intake by changing their activity levels, their location within the enterocyte and by regulating the expression of the encoding genes [4]. They are also controlled by β-adrenergic agonists [5], gastrointestinal hormones such as glucagon-like peptide-2, GLP-2 [6], [7], glucose-dependent insulinotropic polypeptide, GIP, cholecystokinin (CCK) [8], and by leptin [9].

Initially characterized as an adipocyte specific protein controlling body weight and adiposity, leptin is now considered as an hormone with pleiotropic biological effects. This status of leptin is consistent with the production of leptin by various tissues and organs including the stomach. The stomach-derived leptin is rapidly and mainly secreted into the gastric juice after a meal [10] where it is not fully degraded even at pH 2 [10], [11]. The released leptin enters the intestine and is detected in intestinal juices from duodenum to the colon as both free leptin and leptin bound to its soluble receptor Ob-Re [12], [13] as previously reported for plasma leptin. The demonstration that leptin receptors are present all along the small and large intestine [14], [15], are in line with leptin, acting at the luminal side to enhance intestinal absorption of oligopeptides mediated by the proton-dependent PepT1 transporter [14], to increase monocarboxylate transporter MCT-1 butyrate uptake in Caco-2 cells [16], and to inhibit the active component of glucose absorption mediated by SGLT-1 [9]. However, whether the apical GLUT2 transporter [1] which is active during a meal and the fructose GLUT5 transporter can be direct targets for luminal leptin, is unknown.

The aim of the present study was to investigate the effects of luminal leptin (mimicking gastric leptin) on the transport activities and expression of GLUT2 and GLUT5 transporters in the small intestine and to analyse the intracellular mechanisms involved. Since fructose represents an important ingredient in human diets due to the extensive use of high-fructose sweeteners [17], [18], and it has been described as a contributing factor in the metabolic syndrome, we analysed the *in vivo* effects of luminal leptin regulation of fructose absorption on some key indices of lipid and carbohydrates metabolism. Then, we questioned the physiological relevance of the data by determining whether fructose ingestion modulated gastric leptin and whether this leptin is actually entering into the intestinal lumen.

In this study, we demonstrated that luminal leptin, acting from lumen of the intestinal epithelium, increases GLUT2 and GLUT5 transport activities through leptin-receptor coupled to activation of protein kinase C subunit βII (PKCβII) and 5′AMP-activated protein kinase subunit α (AMPKα). We also demonstrated, for the first time, that oral fructose rapidly induces the release of leptin in the gastrointestinal lumen without significantly affecting plasma leptin levels. Moreover, in *vivo* oral leptin-induced stimulation of GLUT5-mediated fructose transport has several consequences: *i)* a significant rise in blood glucose levels associated with changes in mRNA levels of key gluconeogenesis enzymes; *ii)* an increase in circulating levels of triglycerides associated with a reduced transcription of intestinal and hepatic fasting-induced adipocyte factor (Fiaf), encoding a secreted lipoprotein lipase (LPL) inhibitor, and *iii)* enhanced transcription of sterol regulatory element-binding protein-1c (SREBP-1c), acetyl-coA carboxylase isoform 1 (ACC-1) and fatty acid synthase (FAS) in the liver.

## Results

### Luminal Leptin Increases GLUT5-Mediated Fructose Transport

**Figure 1:**
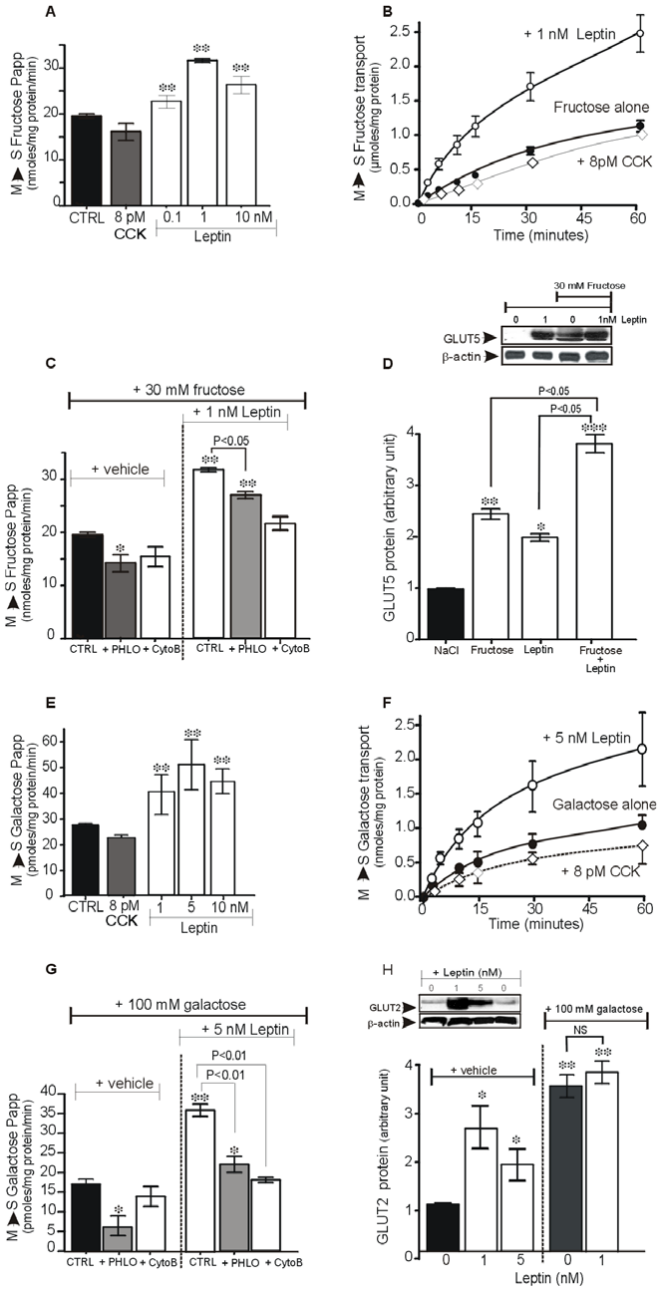
Luminal leptin increases GLUT2 and GLUT5 transport activities. Dose-response effect for luminal leptin stimulation of mucosal *(M)* to serosal *(S)* fructose [30 mM, (**1, A**)] or galactose [100 mM, (**1, E**)] transepithelial transport in the isolated rat jejunal loops *in vitro*. CCK8 (8 pM) was added to the serosal side. Data are expressed as apparent permeability (Papp) and each column represents the mean±SEM of n = 6 rats. **B, F,** Kinetic of luminal leptin stimulation of fructose (**B**) or galactose (**F**) transport in the isolated rat jejunal loops *in vitro*. CCK8 (8 pM) was added in the incubation medium (serosal side). **C, G,** Effects of phloretin (PHLO, 1 mM) and cytochalasin B (CytoB, 20 µM), two GLUT2 inhibitors, on basal and leptin-induced mucosal to basolateral fructose (**C**) and galactose (**G**) transport in the isolated rat jejunal loops *in vitro*. Data are expressed as apparent permeability (Papp) and each column represents the mean±SEM of n = 8 rats. **D, H,** Representative immunoblots of GLUT5, GLUT2 and β-actin proteins in brush-border membranes from rat jejunum treated with or without luminal leptin in association with or without fructose (30 mM) and galactose (100 mM) into the isolated jejunal loop. Results of densitometric analysis are expressed as relative protein levels. *P<0.05; **P<0.01,*** P<0.001 *vs.* NaCl.

Addition of leptin into jejunum loop *in vitro*, induced a concentration-dependent increase in P_app_ of [^14^C]-fructose (fig. 1, A) with a maximal increase occurring with 1 nM leptin (P<0.01 *vs.* CTRL). This transepithelial transport of fructose was rapid and time-dependently increased with 1 nM leptin added into the jejunum (fig. 1, B). Addition of CCK-8 in the serosal side did not significantly affect fructose transport. Under these conditions, luminal addition of leptin had no effect on [^14^C]-mannitol transport (data not shown), indicating that the increase in fructose transport was unlikely to have been caused by a change in passive tissue permeability.

On the other hand, addition of phloretin significantly reduced by 20% (P<0.05) both basal- and leptin-stimulated fructose transport (fig. 1, C) consistent with the involvement of a small component of GLUT2 in fructose transport [3]. The addition of cytochalasin B also reduced basal and suppressed leptin-stimulated fructose transport (fig. 1, C).

The intestinal absorption of sugars is often associated with substrate-induced translocation of transporters to apical membranes [1], [19]. We observed that leptin-stimulated fructose transport was associated with an increased level of GLUT5 protein in the BBM (fig. 1, D). Thus, luminal fructose induced a 2.2-fold increase in the levels of GLUT5 in the BBM (P<0.01 *vs.* CTRL) and when combined with leptin, this level was 3.4 times higher than in controls.

### Luminal Leptin Increases GLUT2-Mediated Galactose Transport

As for fructose transport, addition of leptin into rat jejunum loop *in vitro* induced a concentration-dependent increase in galactose uptake (fig. 1, E). The maximal effect of leptin on apparent permeability (P_app_) of galactose (2-fold increase; P<0.01 *vs*. control) occurred with 5 nM luminal leptin compared to 1 nM for fructose transport. These apparent different sensitivities of GLUT2 and GLUT5 could be related to the high concentration of galactose used in the study taking into account the differences in Km of the two transporters and, we used the dose of 5 nM for the time course studies for galactose transport. In fig. 1F, [^14^C]-galactose transport across the jejunum (mucosal to serosal) significantly increased over time; this effect was enhanced by luminal addition of 5 nM leptin. Addition of CCK-8 at the serosal side (incubation bath) had no significant effect. Moreover, the leptin stimulation of galactose transport was markedly reduced by phloretin and cytochalasin B, two inhibitors of GLUT2 transporter (fig. 1, G) suggesting that GLUT2 transporter is a direct target of leptin. In fig. 1H, we found that luminal leptin significantly increased the levels of GLUT2 at the BBM: with 1 nM leptin, GLUT2 levels increased by 2-fold (P<0.01 vs. control). Galactose (100 mM) induced a 3-fold increase in GLUT2 levels in the BBM and this effect was not further increased by addition of leptin into the jejunum.

### Leptin's Action Is Leptin-Receptor Specific

**Figure 2:**
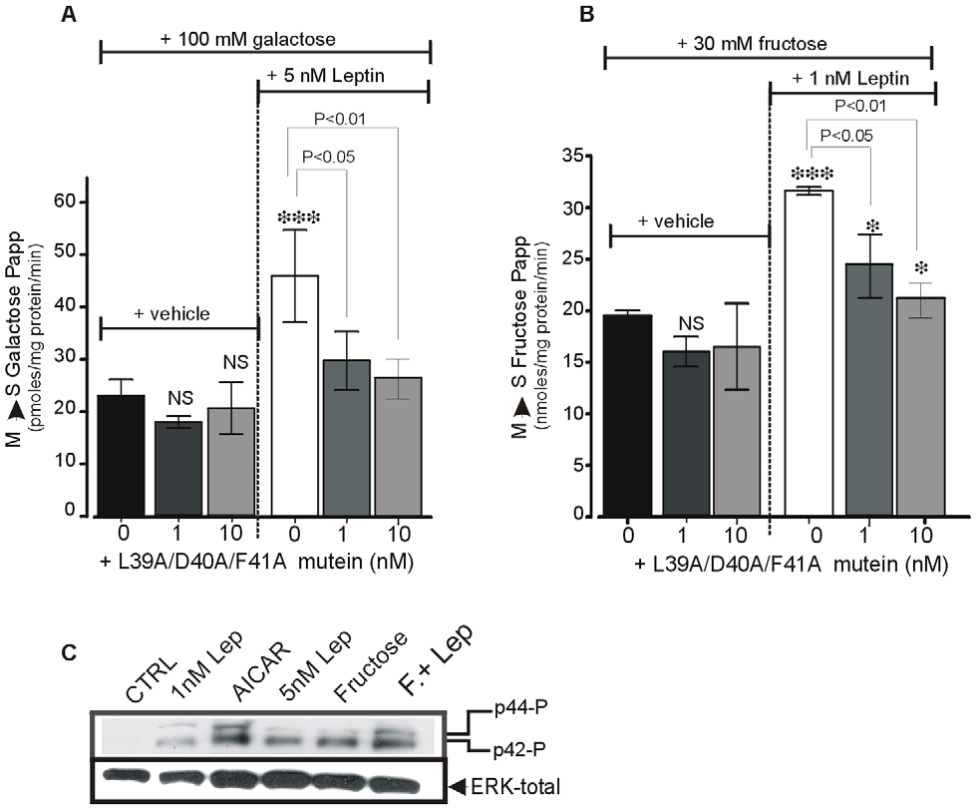
Actions of leptin require active jejunal leptin receptors. Effects of a leptin receptor antagonist, L39A/D40A/F41A mutein on basal and luminal leptin-stimulated mucosal to serosal D-galactose (**A**) and D-fructose (**B**) transport across the rat jejunum. Shown are apparent permeability (Papp) of galactose and fructose across the jejunum. Each column represents mean±SEM for n = 6 rats. Statistical analysis was performed using One-way ANOVA followed by a Tukey Kramer multiple comparisons *P<0.05, ***P<0.001 vs. control. (**C**) Leptin receptors in the jejunum mucosa are functional. Shown are representative immunoblots (4 separate experiments) of phosphorylated ERK-1/2 and total ERK proteins in extracts from isolated jejunum treated with luminal leptin or fructose or both in association. The introduction of leptin into jejunum increased phosphorylation of ERK-1/2. Luminal fructose also increased phosphorylation of ERK-1/2, an effect that was further enhanced by leptin. AICAR, the pharmacological activator of AMPK increased the phosphorylation ERK-1/2.

To address receptor specificity of leptin'effects, we used the murine L39A/D40A/F41A leptin mutein, a recent developed recombinant leptin analog in which alanine was substituted for amino acids expressed in positions 39, 40, and 41 of wild-type leptin. This leptin mutein behaves as an antagonist of leptin receptor [20], [21]. In fig. 2, we found that addition of L39A/D40A/F41A mutein into isolated jejunal loops *in vitro* did not affect basal [^14^C]-galactose (fig. 2, A) or [^14^C]-fructose (fig. 2, B) transport but dose-dependently prevented leptin stimulation of both galactose and fructose transport.

In addition, in rat, the *in vivo* leptin stimulation of GLUT2-mediated galactose transport was associated with a rise of galactose levels in the blood (Text S1, fig. S1). Moreover, addition of L39A/D40A/F41A, a leptin antagonist, completely prevented leptin-induced increase in blood galactose (fig. S1). This indicates that the effects of luminal leptin are dependent upon leptin receptor, consistent with our previous data using leptin-receptor deficient *fa/fa* rats [12], [9].

These leptin receptors were further demonstrated to be functional since they are responsive to luminal leptin alone or combined with luminal fructose (fig. 2, C). Indeed, activation of leptin receptor by introduction of leptin into the lumen of the jejunum loop, increased phosphorylation of ERK-1/2 consistent with the well-known coupling of activated leptin-receptor to MAPkinase signaling pathways. It is noteworthy that luminal fructose increased phosphorylation of ERK-1/2, an effect that was further enhanced by leptin. Similarly, AICAR, a pharmacological activator of AMPK, induced a strong phosphorylation of ERK-1/2 proteins in jejunal mucosa. These data can be compared with the capacity of central fructose to sense via activation of hypothalamic AMPK/malonyl-CoA signaling pathways [22].

### The Leptin Stimulation of Fructose Transport Is Associated with Increase Phosphorylated PKCβII in the BBM

**Figure 3:**
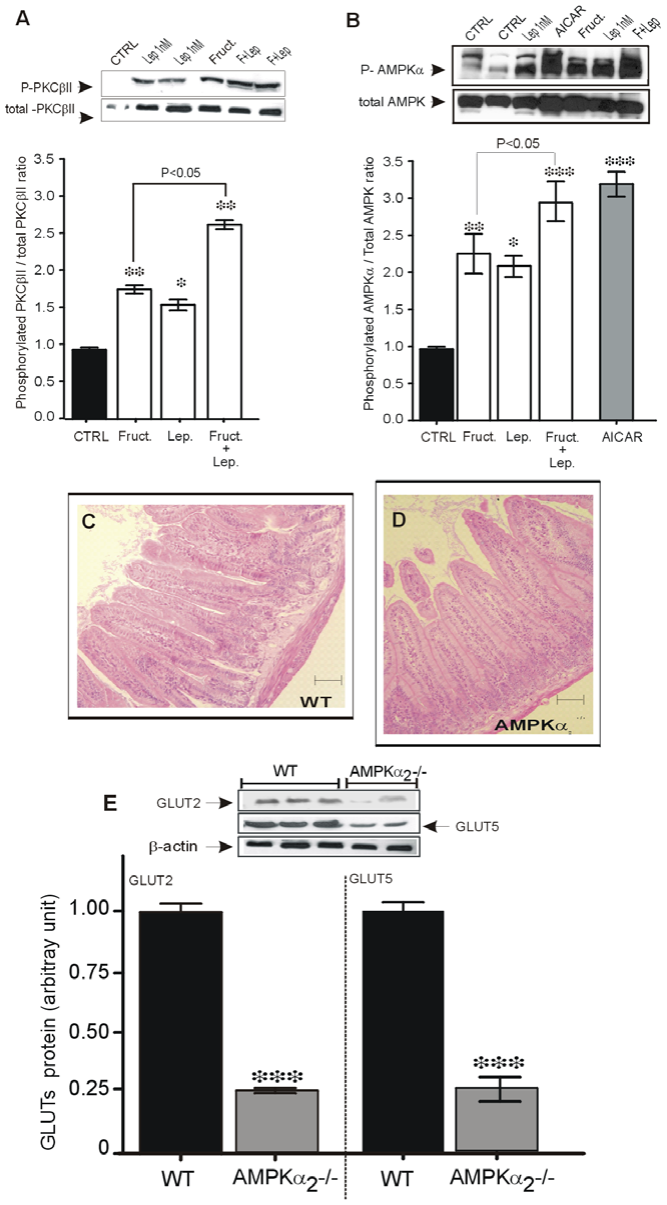
Luminal leptin phosphorylates PKCβII and AMPKα in the jejunum. **A,** A representative immunoblot (5 different preparations of BBM) of phosphorylated and total PKCβII from rat jejunum loops treated with or without luminal leptin in association with or without 30 mM fructose. The data of densitometric analysis of the blots are expressed as a ratio of phosphorylated PKCβII to total PKCβII. **B,** a representative immunoblot (4 separate experiments) of phosphorylated and total AMPK from mucosa extracts of rat jejunum loops incubated with or without (CTRL) 1 nM leptin in association without or with 30 mM fructose. 5-aminoimidazole-4-carboxamide riboside (2.5 mM AICAR) was used as control. Data of densitometric analysis are expressed as a ratio of phosphorylated AMPKα to total AMPK. *P<0.05, ** P<0.01 and *** P<0.001 *vs.* CTRL. **C, D,** histological analysis of jejunum sections from AMPKα_2_−/− and WT mice stained with hematoxylin-eosin (scale bar = 20 µm). **E,** representative immunoblots of GLUT2 and GLUT5 proteins in extracts from jejunum mucosa from AMPKα_2_−/− and WT mice. Data of densitometric analysis of the blots are expressed as relative protein levels. Each column represent the mean±SEM of n = 5 mice in each group. *** P<0.001 vs WT.

Previous studies have shown that the increase in recruitment of sugar transporters to the BBM requires activation of the βII-isoenzyme of protein kinase C [23]. In this study, luminal fructose increased phosphorylation of PKCβII in the BBM, an effect which was further enhanced by addition of 1 nM leptin into the jejunum (fig. 3, A). These data might indicate that leptin enhances fructose transport, by increasing phosphorylation/activation of PKCβII and by recruiting more GLUT5 proteins into the BBM.

### Leptin-Stimulated Fructose Transport Results in a Concurrent Increase of AMPK Phosphorylation

AMPK has been previously implicated in the regulation of glucose uptake (*review in* [24]). We examined whether AMPK can mediate the action of leptin in the intestine. We found that luminal leptin alone or in association with fructose significantly increased phosphorylation of the catalytic α subunit of AMPK (fig. 3, B). Indeed, fructose or leptin (1 nM) added into the lumen increases phosphorylation of AMPKα by 2-fold as compared to controls (fig. 3, B). Interestingly, treatment with a combination of fructose and leptin gave levels of phosphorylated AMPKα 3-fold higher than those observed in controls (P<0.05). This increased phosphorylation was similar to that induced by AICAR, a pharmacological activator of AMPK. These data argue for the involvement of the α subunit of AMPK in mediating the effects of leptin.

We next used AMPKα_2_ knockout (AMPKα_2_−/−) mice to further study the role of α_2_ subunit of AMPK in the effects of leptin. Histological analysis showed that there was no difference in the architecture of jejunum mucosa between WT and AMPKα_2_ −/− mice (fig. 3, C, D). However, Western blot analysis of extracts from jejunum of AMPKα_2_ −/− mice (fig. 3, E) showed that levels of GLUT2 and GLUT5 proteins were three times lower than in the jejunum of WT mice (P<0.001). In parallel, SGLT-1 transporters were increased in AMPKα_2_−/− by 2-fold as compared to WT mice (Text S1, fig. S2). The transport activity of SGLT-1 was determined in Ussing chamber. The addition of 10 mM glucose into the mucosal bath led to a rapid and significant rise in the short circuit current (*Isc*) which was 2.3 times higher in jejunum from AMPKα_2_−/− mice than in jejunum from WT mice (P<0.01). These data indicate that leptin controls the activity/expression of sugar transporters in the small intestine through mechanisms requiring the α_2_ subunit of AMPK.

### Oral Leptin Increases GLUT2 and GLUT5 mRNA Levels in the Jejunum

We have determined whether administration of 3 ng/g leptin to mice by gavage either alone or with fructose, could replenish the cytosolic pool of transporters by increasing the levels of their mRNA levels. This dose of leptin was chosen from our pilot experiments showing that the peptide given by gavage generates levels of leptin similar to those detected in the stomach after meal. Moreover, 15 min after gavage, 70% of the peptide was detected in the small intestine representing a concentration sufficient to activate leptin receptors.

**Figure 4:**
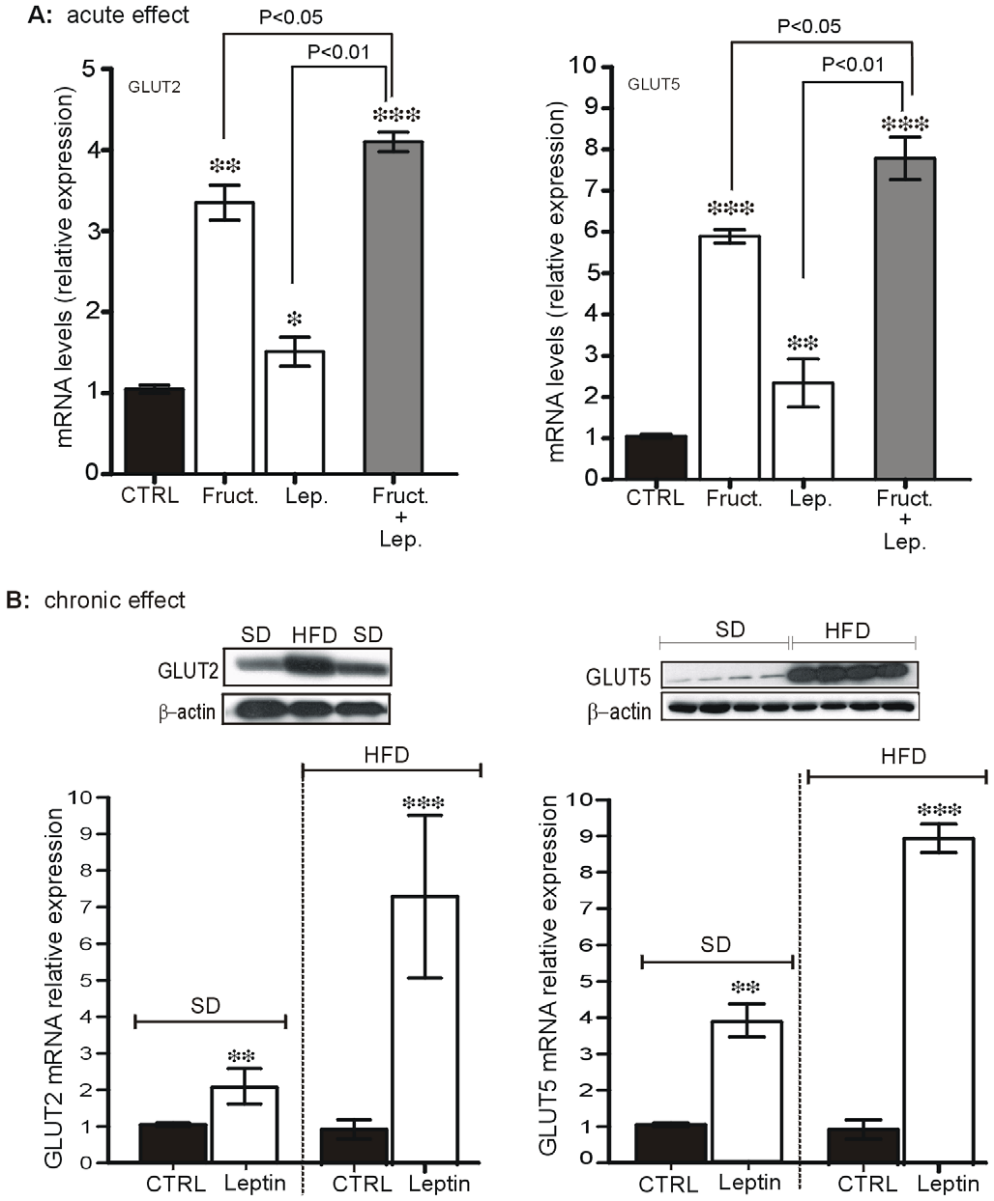
Acute and chronic leptin administration increase basal and fructose-stimulated GLUT2 and GLUT5 mRNA. **A,** for acute studies, 16-hour deprived fasted mice receiving oral administration of saline (CTRL), 3 ng/g murine leptin, fructose (2 g/kg) or fructose in combination with leptin. They were sacrified 4 hours later, and total RNA was extracted from the jejunum mucosa for qRT-PCR analysis in duplicate using specific oligonucleotides for GLUT2 and GLUT5 genes. Results are means±SEM for 8 mice in each group. **B,** for chronic studies, mice fed a standard diet (SD) or a high-calorie diet (HFD), received saline (CTRL) or 3 ng/g leptin, administered daily by gavage for 7 days. Mice were killed on day 8 and total RNA was extracted from jejunum mucosa for qRT-PCR analysis. Results are means±SEM for 8 mice in each study and each group. * P<0.05; ** P<0.01 and *** P<0.001 vs CTRL. **B insert,** representative immunoblots of GLUT2 and GLUT5 proteins from jejunum extracts of mice fed a SD and a high-calorie diet (HFD) showing increased amount GLUT2 and GLUT5 proteins when mice were fed a HFD in comparison to those fed a SD.

As shown in fig. 4A, mice receiving leptin by gavage, had 1.6-fold and 2 -fold higher levels of GLUT2 and GLUT5 mRNAs in the jejunum respectively, than control mice receiving saline. Oral load of fructose increased by 3-fold (P<0.01 vs CTRL) and 5-fold (P<0.01 vs CTRL) GLUT2 and GLUT5 mRNA levels, respectively, consistent with previous reports [25], [26]. Leptin given together with oral fructose significantly enhanced this effect, increasing GLUT2 mRNA levels by a factor of 4 (*vs.* 3-fold for fructose) and those of GLUT5 by a factor of 7 (*vs.* 5-fold).

This acute effect of leptin was still observed after 7 days of daily treatment of the mice with leptin (fig. 4, B) and was exacerbated in mice fed on a HFD. In mice fed with HFD for 7 days in comparison to those fed with ND, the levels GLUT2 and GLUT5 proteins are significantly increased (fig. 4, B insert). Moreover, treatment of HFD mice with leptin by gavage, led to a dramatic increase in the mRNA levels with a 6-fold increase in GLUT2 mRNA (P<0.001) and a 9-fold increase in GLUT5 mRNA (P<0.001) in vehicle-treated mice (fig. 4, B).

### The Actions of Leptin Are Followed by Changes in Blood Fructose and Glucose Levels

**Figure 5:**
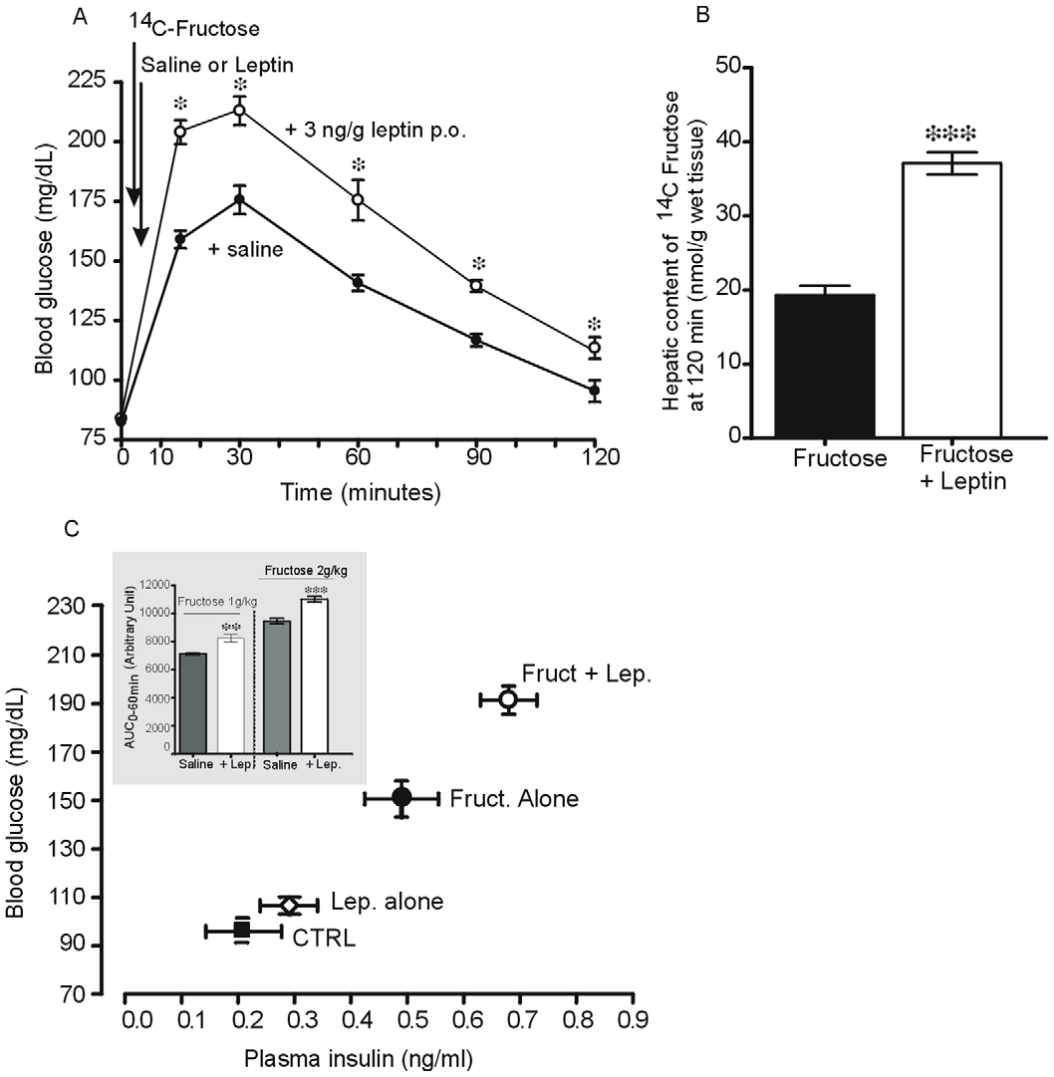
Luminal leptin increases fructose-induced hyperglycaemia and hepatic content of fructose. Changes in plasma glucose levels (**A**) and in hepatic content of fructose (**B**). A solution of fructose (2 g/kg) containing radiolabelled [^14^C]-fructose was given by gavage without (control) or with 3 ng/g leptin to 16-hour fasted mice. Before starting the oral fructose tolerance test (OFTT), blood samples were taken from the tail and glucose levels was determined using Accu-Chek. At 120 minutes, the radioactivity in liver was counted and used to calculate the hepatic content fructose. **C,** changes in blood glucose levels as a function of plasma insulin levels. Fifteen minutes after oral administration of fructose (2 g/kg) without (control) or with 3 ng/g leptin to 16-hour fasted mice, blood was collected as described in *Materials and Methods* for determination of plasma glucose and insulin levels. **C insert,** are the measured area under curves at time 60 min after oral load of fructose (1 or 2 g/kg) without or with 3 ng/g leptin. Each column represents the mean±SEM of n = 8 mice for saline and leptin, n = 12 for each amount of fructose and fructose+leptin. * P<0.05 *vs*. saline; # P<0.05 *vs* fructose.

As expected, oral administration of radiolabelled ^14^C-fructose induced a time-dependent increase of ^14^C fructose in blood in parallel with glucose (fig. 5, A). When fructose was given in combination with leptin, a rapid and significant increase in fructose-induced hyperglycaemia occurred as soon as 15 min. The +20% increase (P<0.05 *vs.* fructose) reached at 15 min was maintained over the 120 min period. At that time, the amount of ^14^C fructose detected in the liver was significantly 2-fold higher with fructose in combination with leptin than with fructose alone (fig. 5, B).

This enhancement of fructose-induced glycaemia by leptin given orally was dependent on the amount of ingested fructose. Indeed, when the same dose of leptin (3 ng/g) was combined with low amount of fructose (1 g/kg), an increase in fructose-induced hyperglycaemia was also observed but, at levels significantly lower than those levels seen with 2 g/kg fructose (data not shown). The areas under curves for both low (1 g/kg) and the high (2 g/kg) fructose (fig. 5, C insert) were significantly increased when leptin was given in association with fructose. Plasma insulin levels, 15 min after administration of fructose alone or in combination with leptin, significantly increased in parallel with the levels of glucose (fig. 5, C). The increase in circulating levels of glucose and in liver fructose content after oral fructose either alone or in combination with leptin, suggests a possible enterocyte and/or liver production of glucose. To verify these possibilities, we quantified mRNA levels of some key enzymes involved in gluconeogenesis.

### Changes in mRNA of Key Gluconeogenesis Enzymes

**Figure 6:**
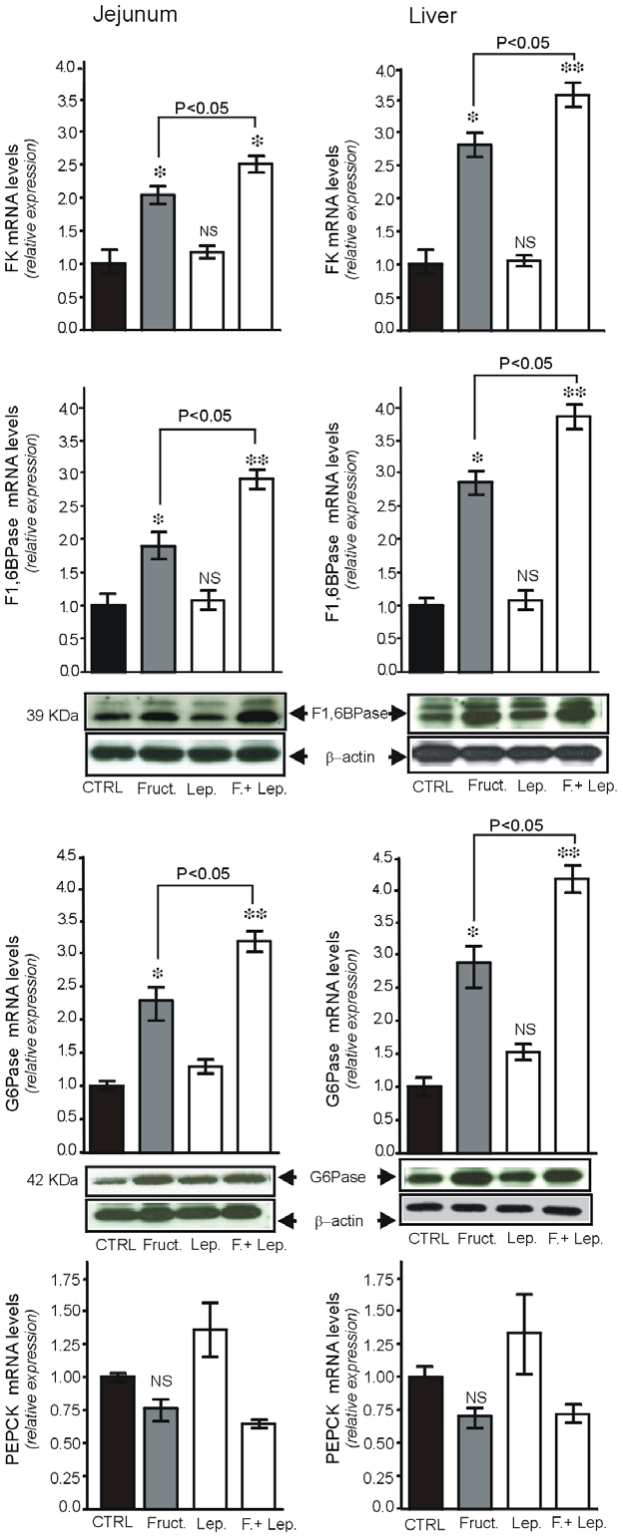
Leptin given orally potentiates fructose-induced mRNA levels of enzymes involved in glucose metabolism in intestine and the liver. Total RNA was extracted from jejunum mucosa and the liver, 4 hours after oral administration of saline (CTRL) or 3 ng/g leptin, oral fructose (2 g/kg) or both, in fasted mice. Q-RT PCR analysis was performed in duplicate using specific oligonucleotides for fructokinase, F1,6BPase, G6Pase and PEPCK genes, left columns: jejunum, right columns: liver. **Inserts** are representative immunoblots of F1,6BPase, glucose-6-phosphatase (G6Pase). Results are means±SEM for 6 mice in each group. * P<0.05 and ** P<0.01 *vs.* CTRL.

Previous studies have shown that, together with increased GLUT5 mRNA, the expression of genes encoding gluconeogenic enzymes also increased significantly after luminal fructose [27]. We found that oral load of fructose induced a significant increase in the levels of FK, F1,6BPase and G6Pase mRNAs and did not significantly affect PEPCK mRNA levels in jejunum and the liver (fig. 6). Leptin given by gavage did not significantly modify mRNA levels of these enzymes. When combined with oral fructose, leptin potentiated fructose-induced increase in the levels of FK, F1,6BPase and G6Pase mRNA both in the intestine and the liver (fig. 6). These changes in F1,6BPase and G6Pase mRNA levels paralleled those of their protein levels in the intestine and the liver (fig. 6 insert).

### Changes in mRNA levels of Some Enzymes of Lipid Metabolism

**Figure 7:**
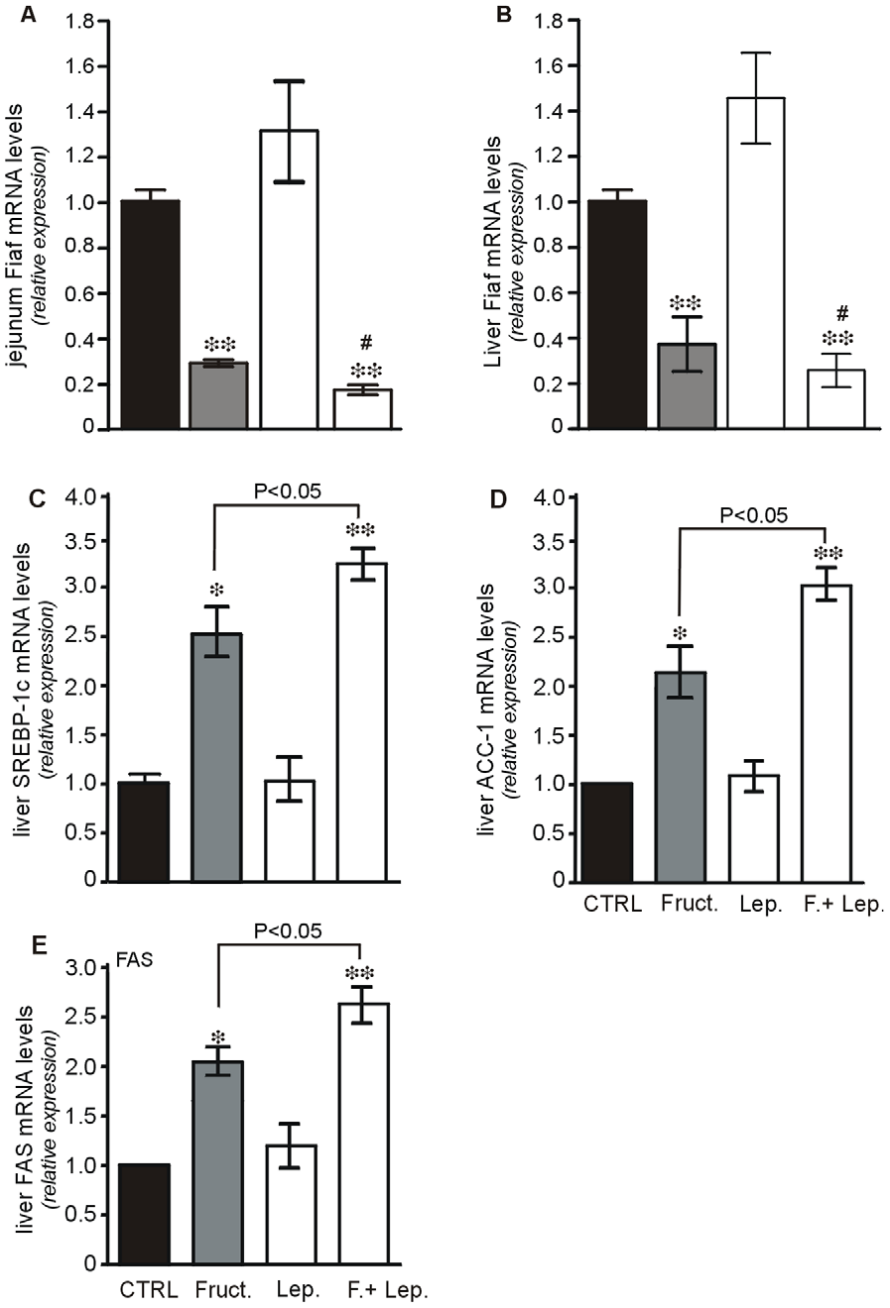
Changes in Fiaf, hepatic SREBP-1c, ACC-1 and FAS mRNA in response to oral administration of fructose or leptin or both. Total RNA was extracted from jejunum mucosa and the liver, 4 hours after oral administration of saline (CTRL), leptin (3 ng/g), oral fructose (2 g/kg) or both, in fasted mice. QRT-PCR analysis was performed in duplicate using specific oligonucleotides targeting genes encoding (**A, B**) fasting-induced adipocyte factor (Fiaf), (**C**) liver SREBP-1c, (**D**) liver ACC-1 and (**E**) liver FAS. Results are means±SEM for 6 mice in each group are expressed as mRNA levels relative expression. * P<0.05; ** P<0.01 *vs* CTRL.

In addition to the mild hyperglycaemia induced by fructose, the levels of blood triglycerides were significantly increased (Table 1). Thus, we first questioned whether oral load of fructose without or with leptin could modify the expression of Fasting-induced adipocyte factor (Fiaf). The Fiaf gene encodes a secreted lipoprotein lipase (LPL) inhibitor that is also produced in intestinal epithelium and the liver [28]. We found unexpectedly that oral load of fructose induced a significant 4-fold (P<0.01 vs. CTRL) and 3-fold (P<0.01 vs. CTRL) reduction in intestinal and hepatic Fiaf mRNA levels, respectively (fig. 7, A, B). When leptin alone was given orally, there were no significant changes in the levels of Fiaf mRNA both in intestine and the liver. However, when combined with fructose, leptin exacerbated fructose-induced decrease in Fiaf mRNA levels. This suppression of Fiaf mRNA levels is likely to result in an increase in LPL activity, an enzyme mediating the rate-limiting step for triglyceride-derived fatty acid production.

**Table 1:** Plasma levels in 18-hour fasted mice receiving orally saline (CTRL), fructose (2 g/kg), leptin (3 ng/g) or fructose with leptin.

	CTRL	Fructose 2 g/kg	Leptin 3 ng/kg	Fructose + Leptin
Plasma leptin (*ng/mL)*	1.50±0.35	2.85±0.37*	1.42±0.20	2.81±0.21
Plasma insulin *(ng/mL*	0.21±0.02	0.50±0.13*	0.27±0.05	0.59±0.12*
Blood glucose *mmol/L*	2.60±0.25	4.85±0.25	3.71±0.28*	5.78±0.22**#
Blood triglycerides *mmol/L*	0.95±0.06	1.32±0.16*	0.60±0.04 *	1.45±0.13
HDL cholesterol *mmol/L*	1.54±0.22	1.24±0.32	1.57±0.11	1.37±0.20
Total cholesterol *mmol/L*	1.88±0.25	3.21±0.28*	2.07±0.21	2.97±0.24*

Mice were killed 4 hours after gavage, blood was collected, processed as described *in* *Material and Methods* and used to determine biochemical parameters.

Data are expressed as mean±SEM of n = 8 mice per group. One-way ANOVA followed by a multiple comparisons Tukey t-test were used for statistical analysis.

* P<0.05,

** P<0.01 *vs.* CTRL;

#P<0.05 *vs.* fructose alone.

**Figure 8:**
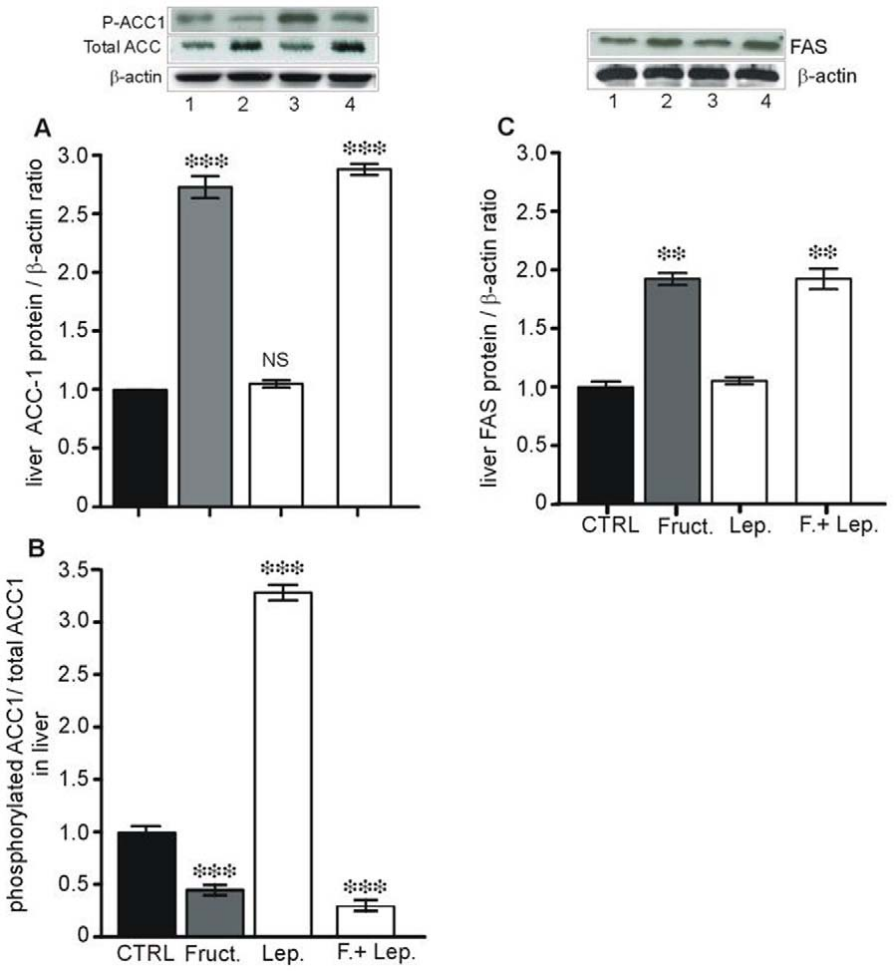
Changes in hepatic ACC-1 and FAS proteins in response to fructose, leptin or both given orally. Proteins were extracted from jejunum mucosa and the liver, 4 hours after oral administration of saline, leptin (3 ng/g), oral fructose (2 g/kg) or both, in fasted mice. Representative immunoblots of phosphorylated ACC-1, total ACC-1, FAS and β-actin protein in liver extracts. Lane 1: control; lane 2: Fructose 2 g/kg: lane 3: leptin 3 ng/g; lane 4: fructose in combination with leptin. Results of densitometric analysis are expressed as mean±SEM of 6 mice in each group. ** P<0.01 and *** P<0.01 *vs.* CTRL.

Second, given that fructose consumption can promote lipogenesis in liver, the main site of fructose metabolism, the mRNA levels of SREBP-1c (a transcription factor targeting lipogenic genes) and two lipogenic enzymes were examined specifically in the liver. As shown in fig. 7C, levels of SREBP-1c mRNA were twice as high in mice receiving oral fructose alone than in controls (P<0.01 *vs.* CTRL). This effect was more potent when combined with leptin (P<0.05 *vs.* fructose alone). In parallel, hepatic ACC-1 and FAS mRNA levels (fig. 7, D, E) significantly increased and this increase was boosted when leptin was added (P<0.05 vs. fructose). The changes in the levels of ACC-1 and FAS mRNA paralleled those of ACC-1 and FAS proteins (fig. 8, A, C).

To further assess the impact of increased expression of ACC-1, phosphorylation of the enzyme was determined as an index of activity. As shown in fig. 8B, the phosphorylation of ACC-1 protein significantly decreased by 2-fold after oral load of fructose and by 3-fold when combined with leptin, indicating an increased ACC-1 activity. It is noteworthy that oral leptin alone significantly increased by 3-fold the phosphorylation of ACC-1 indicating that leptin given orally inactivates ACC-1 in the liver.

### Oral Fructose Rapidly Increases Luminal Content of Leptin in Gastric Juice without Affecting Plasma Leptin Levels

**Figure 9:**
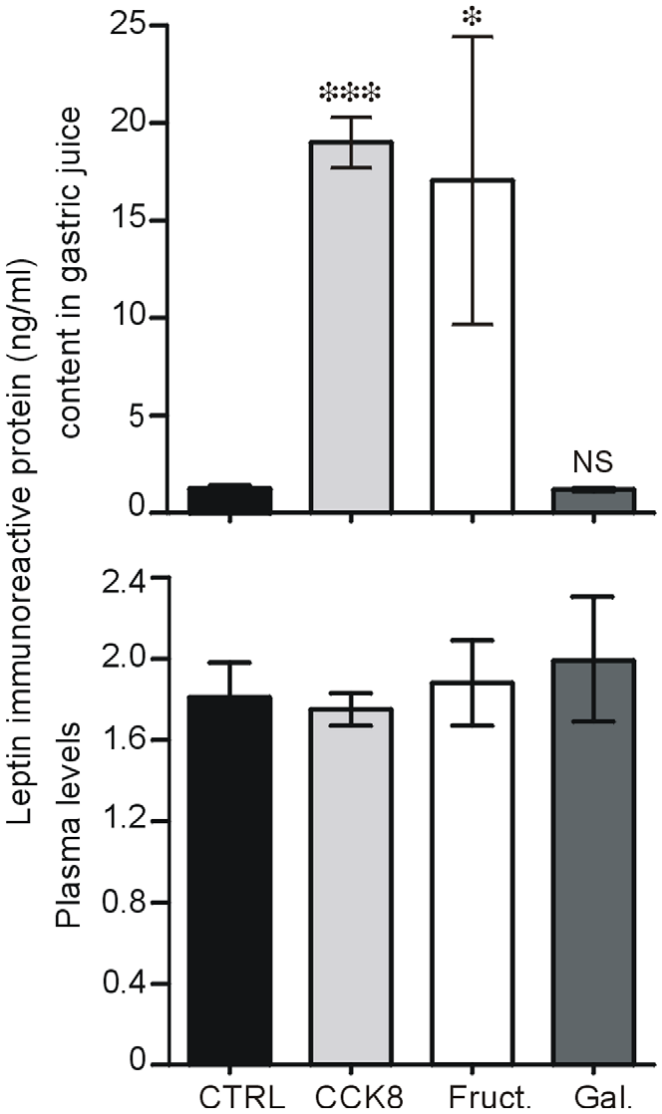
Release of leptin in gastric juice in response to oral load of fructose or galactose. Changes in the amount of leptin released in the gastric juice and in plasma in mice fasted overnight and receiving an oral load of saline (CTRL), fructose [Fruct. 2 g/kg)], galactose (Gal. 6 g/kg) or intraperitoneal injection of 10 ng/g CCK-8. Leptin immunoreactivity was determined by RIA in 15-min collected juice from the stomach as described *in Materials & Methods.* Data are expressed as the mean±SEM of *n* = 6 in control group and n = 5 in each other group. *P<0.05 and ***P<0.01 *vs* CTRL.

To determine the physiological relevance of these results, we analyzed the effects of oral fructose on luminal contents of leptin from stomach and the small intestine in fasted mice (fig. 9). Unexpectedly, oral load of fructose but not galactose, resulted in a dramatic 10-fold increase in basal leptin content in gastric juice (P<0.05 vs CTRL). This effect was rapid and seen as early as 15 min. This rapid increase was similar to that induced by i.p. administration of CCK-8 (used as a positive control) in agreement with our previous findings in rats [10]. It is noteworthy that during this 15-min period, the increase in gastric leptin output in response to fructose was not associated with significant change in plasma leptin levels (fig. 9). However, 4 hours after oral fructose, the levels of plasma leptin were significantly increased (Table 1) probably reflecting the contribution of fat tissues.

## Discussion

The small intestine is the primary site of absorption of dietary sugars. Thus, a better understanding of the regulatory mechanisms involved in their entry and exit from intestinal epithelium to blood, is of great interest. In this combined *in vivo* and *in vitro* approaches, we showed that intestinal GLUT2 and GLUT5 transporters are direct targets of luminal leptin detected in the intestinal lumen and originating from the stomach. Indeed, introduction of exogenous leptin into intestinal lumen resulted in an up-regulation of transepithelial transport of fructose, a concurrent potentiation of circulating levels of glucose, and an increase in fructose content in the liver. These effects were accompanied by increased circulating levels of triglycerides, suppression of Fiaf expression both in intestinal epithelium and the liver, and by induction of hepatic SREBP-1c transcription factor, targeting genes in the fatty acid biosynthesis pathway. These findings may have physiological implications. Thus, we demonstrated for the first time that oral load of fructose, but not galactose rapidly induces release of gastric leptin which enters the intestine at a concentration compatible with the activation of leptin receptors. Collectively, these data sustain that in the small intestine, a positive regulatory control loop exists between gut leptin and two transporters: the specific GLUT5 fructose transporter and the apical GLUT2 which is active in conditions of high concentrations of glucose/galactose and which has a minor participation in fructose transport. This regulatory loop appears to be connected to metabolic functions in the liver.

Firstly, we showed that mucosal leptin increases GLUT2 and GLUT5 transporter activity as well as insertion of GLUT2 and GLUT5 in the brush-border membrane (BBM). These changes were leptin-receptor specific since they were blocked by leptin antagonist and correlated with the phosphorylation/activation of PKCβII at the BBM. This activation of PKCβII enhanced the insertion of GLUT2/5 and reduced the insertion of the energy-dependent SGLT1 transporters into the brush-border membrane, consistent with previous data [2], [9], [23]. The findings have clear physiological implications: immediately after a meal, a high local concentration of sugars is generated at the surface of the brush-border by disaccharidases. The released gastric leptin [10] enters the lumen of the intestine [12], [30], where it may then up-regulate GLUT2/GLUT5 transporter activity to match dietary intake. The intracellular mechanisms of such a rapid regulation of absorption by luminal leptin involve the activation of AMPKα which is known to play a central role in regulating glucose uptake (*review in* [24]). In this context, we showed that luminally acting leptin phosphorylates/activates AMPKα in the intestine consistent with previous data reporting similar findings in other biological systems *i.e*. liver, hypothalamus, muscles [29], [31]–[33]. Interestingly, we found that leptin given orally can boost the fructose activation of AMPKα leading to increased GLUT2/GLUT5 transport activities, indicating that these transporters are responsive to AMPK activation [34]. We further elucidated a role of AMPK in the leptin regulation of intestinal hexoses transporters by using AMPKα_2_ KO mice [29]. We found that deletion of the catalytic subunit α_2_, which does not alter the architecture of the jejunum, potently lowered levels of GLUT2 and GLUT5 and elevated levels of SGLT1. This increase in SGLT1 levels correlated with an increase in its activity, as measured by glucose-induced *Isc* using the Ussing chamber (fig. S1), and might represent a compensatory effect in the process of sugar absorption. Alternatively, the reduced expression of GLUT2/GLUT5 and the increased SGLT1 expression in AMPKα_2_ KO mice could reflect a differential regulation of these transporters by AMPKα_2_ as reported by Walker and coworkers [34] showing that activation of AMPK results in an up-regulation of GLUT2 and a concurrent down-regulation of SGLT-1. Moreover, in the jejunum of WT mice, luminal leptin inhibited SGLT1-mediated glucose transport which is in line with our previous findings [9]. However, we did not observe this effect in AMPKα_2_ KO jejunum, suggesting that leptin acting from the lumen of the intestinal epithelium requires the catalytic α_2_ subunit of AMPK for this inhibitory effect. Our data can be compared to previous findings showing that activation of AMPK in murine jejunum tissues, increases glucose uptake which can be attributed to an increased amount of GLUT2 in the BBM [34].

On the other hand, *in vivo* luminal fructose increases GLUT2 and GLUT5 mRNA levels as reported earlier [4], [27] and this effect is potentiated by leptin given orally. This suggests that luminal leptin can replenish the pool of the transporters by inducing gene transcription. The *in vivo* dose of leptin was comparable to the doses detected in gastric juice (0.9 to 1.2 nmol/L) in response to fructose as well as the levels of leptin detected in intestinal juice (0.7 nmol/L). These concentrations of leptin can activate leptin receptor [35], [36]. Our demonstration that the actions of leptin require active jejunal leptin receptors, led us to propose a two-step mode of action for luminal leptin regulation of sugars transport, involving a rapid recruitment of GLUT2/GLUT5 to the BBM to deal with the inflow of dietary sugars during a meal, followed by transcription of the corresponding genes, allowing the reconstitution of the cytosolic pool of transporters. These data are summarized in fig. S3.

Notably, the fructose-induced increase in GLUT5 mRNA levels markedly increased when mice fed with a HFD received chronically leptin given orally. This suggests that gut leptin might be a key molecule for the enterocyte in adapting the levels of carbohydrate transporters to the diet. This is in line with recent observations showing that, on an already potent obesity-inducing hypercaloric diet, fructose induces leptin-resistance leading to pronounced susceptibility to increased weight gain [37], [38].

This leptin up-regulation of GLUT2/GLUT5 affects *in vivo* circulating levels of glucose and triglycerides. Indeed, oral leptin enhanced fructose-induced mild hyperglycaemia and the size of this effect is dependent on the amount of fructose absorbed. As a consequence of increased hyperglycaemia, plasma insulin levels increased to adapt glucose uptake by the different tissues. Moreover, all the changes occurring after oral leptin are not associated with changes in plasma leptin levels, indicating that the primary site of action of oral leptin is within the gut mucosa.

In addition to observing the increase in plasma glucose levels after an oral load of fructose, the majority of the absorbed fructose appears in the blood and liver, the main site of fructose metabolism. These findings were consistent with data from studies in rodents and humans [39]. Thus, the increased plasma glucose could originate from fructose metabolism in the liver and possibly from enterocytes. Fructose is metabolised mainly in the liver through two alternative pathways: the fructose 1-phosphate pathway, involving fructokinase, and/or the fructose 6-phosphate pathways. In the enterocyte, fructose is metabolised by fructokinase through the fructose 1-phosphate pathway. As for the liver and kidney, the small intestine expresses gluconeogenesis genes such as those encoding G6Pase and PEPCK [40]. The transcription of these gluconeogenic genes is tightly controlled in the liver by substrates and various hormones [41]. Here, we demonstrate a potentiation by oral leptin of fructose-induced mRNA levels of FK-, F1,6BPase- and G6Pase-encoding genes in the liver and the jejunum. The observed increase in these enzymes mRNA is causally linked to the amount of fructose absorbed; indeed oral leptin alone fails to affect the levels of these enzymes. Thus, as in the liver, glucose produced by the enterocyte may contribute partly to fructose-induced hyperglycemia consistent with a neoglucogenic status for the small intestine [42].

The leptin stimulation of GLUT5 fructose transport is associated with the unexpected reduction of the levels of Fiaf mRNA in both the intestine and the liver. Fiaf is a gene which encodes a physiologically relevant circulating inhibitor of LPL [28], an enzyme that promotes storage of triglycerides [43]. This reduction of intestinal and hepatic levels of Fiaf mRNA, is critically dependent upon the amount of absorbed fructose. Indeed, leptin alone given orally enhances Fiaf mRNA levels, implying that fructose is the primary signal controlling the levels of Fiaf mRNA. The suppression of Fiaf may increase LPL activity and thus activates storage of triglycerides in tissues such as the liver. Our data are reminiscent of those recently demonstrating an enterocyte-specific suppression of Fiaf by the gut microbiota leading to induction of *de novo* hepatic lipogenesis, probably through the increased conversion of dietary polysaccharides into monosaccharides [44]. The fact that leptin potentiates the fructose induction of SREBP-1c, a transcription factor targeting lipogenic genes in the liver [45], [46], strengthens this hypothesis. Interestingly, this leptin potentiation of fructose-induced SREBP-1c was associated with increased expression and activation of ACC-1, the key regulatory enzyme of fatty acid biosynthesis. The activation ACC-1 is expected to elevate the levels of malonyl-CoA in the liver. The concurrent increased expression of hepatic FAS which plays a central role in *de novo* lipogenesis, catalyzing reactions steps in the conversion of malonyl-CoA to palmitate [47], [48], may also indicate increased levels of palmitate. The surprising capacity of leptin given orally to phosphorylate/inactivate ACC-1 in the liver argues for a beneficial effect on the liver. This effect of gut leptin is quite different from the results showing that intracerebroventricular injection of leptin activates hypothalamic ACC, up-regulates the levels of malonyl-CoA and concomitantly inhibits AMPK [49]. We have no clear explanation for these discrepancies but undoubtedly they need to be clarified. The effects of leptin given orally on hepatic metabolic functions could be of great importance in terms of pharmacological manipulation of the expression of these key enzymes, but the mechanisms involved need to be elucidated. Actually, we cannot exclude that leptin given by oral route, could enter the liver in sufficient quantity to affect the hepatic expression of genes encoding key metabolic enzymes. Indeed, we demonstrated that oral fructose but not galactose, triggers a rapid and dramatic secretion of gastric leptin in gastric juice which is found at levels compatible with activation of leptin receptors in the intestinal lumen. These findings are of great interest in light with the recent data demonstrating that consuming fructose-sweetened, and not glucose sweetened, beverages increases *de novo* lipogenesis and increases visceral adiposity in overweight and obese adults [50].

**Figure 10:**
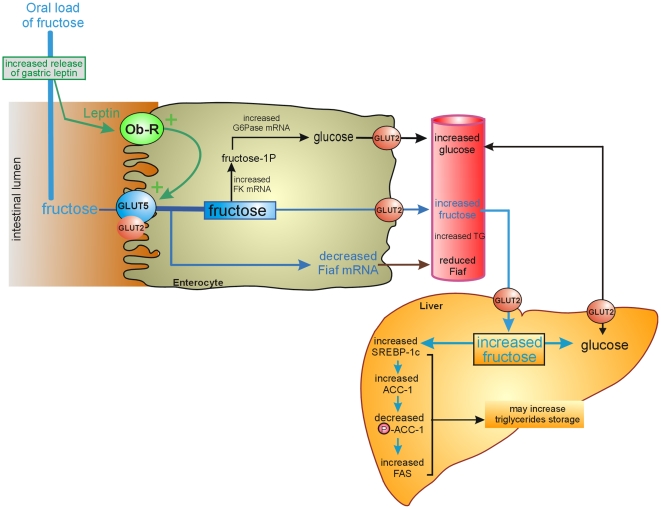
Schematic view of the regulatory control loop between gut leptin and fructose transporters. Ingestion of fructose increased the release of gastric leptin which enters together with fructose the intestine. The concentrations of leptin detected in intestinal juice are suitable with activation of leptin receptors. This luminal leptin operates through leptin receptor (Ob-R) to increase the clearance of fructose from the intestinal lumen predominantly via the brush border GLUT5 transporter. Fructose and glucose exit the enterocyte through the basolateral GLUT2 transporter into the blood and are delivered to various organs including the liver. Fructose enters into the liver, induces SREBP-1c a transcription factor targeting genes in the lipogenic program. This cluster of events which are amplified by gut leptin is likely to provide the mechanism for rapidly acquired adiposity. FK: fructokinase, G6Pase: glucose-6-phosphatase, Fiaf: Fasting-induced adipocyte factor, TG triglycerides, SBREP-1c: Sterol Regulatory Element Binding Protein-1c; ACC-1 Acetyl-coA Carboxylase isoform 1, FAS, fatty acid synthase.

Our data sustain the existence of a positive regulatory control loop between gut leptin and fructose, in which fructose triggers the release of gastric leptin which in turn up-regulates GLUT2/GLUT5 leading to increase fructose absorption and changes in some metabolic functions in the liver. We have summarized these intestine-liver cross talks in fig. 10. This regulatory control loop appears to be a new mechanism (possibly pathogenic) by which fructose consumption rapidly becomes highly lipogenic and deleterious by increasing adiposity. Although rodents may not have identical metabolic pathways to those of humans, our findings provide basis for introducing further nutritional recommendations relative to excess consumption of fructose.

## Materials and Methods

### Animals

Animals used for our experiments were male Wistar rats, C57BL/6J mice 6-8 weeks old (Elevage Janvier, Le genest-St-Isle, France) and AMPKα_2_ catalytic subunit gene knockout (*AMPKα_2_^−/−^*) 10–12 weeks-old mice [29]. All procedures were performed in accordance with the principles and guidelines established by the European Convention for the Protection of Laboratory concerning care and the use of laboratory and with the authorization order N° B75-18-02 delivered by Ministry of Agriculture and Prefecture of Paris, France.

### Transport of Galactose and Fructose in the Rat Jejunal Loop *In Vitro*

The animals were killed, and the transport was monitored using the *in vitro* jejunal loop model. Briefly, a 5-cm segment of jejunum was filled with a Krebs buffer pH 6 containing 100 mM D-galactose or 30 mM fructose added with 0.1 µCi/mL [D-^14^C]-galactose or [^14^C]-fructose (specific activity 57 and 267 mCi/mmoL, respectively) in absence and the presence of murine leptin (PeproTech Neuilly/Seine France). CCK-8 (Sigma Chemicals, St Louis Mo) was added in the bath solution (serosal side) as a positive control. The effect of leptin on transport of [^14^C]-mannitol as non-transported substrate was also determined.

In experiments in which phloretin, cytochalasin B or L39A/D40A/F41A mutein (1, 10 nM) an antagonist of leptin-receptor [20] were used, the jejunum segments were preincubated for 5 min before addition of galactose or fructose solution. The incubation medium was sampled and radioactivity was counted. Apparent permeability (P_app_) was used to assess mucosal to serosal transport according to the following equation P_app_ = (dQ/dt).(V/Q_0_.A) where V is the volume of the incubation medium, A is the area of the loop, Q_0_ is the total radiolabelled galactose or fructose introduced into the loop and dQ/dt is the flux across the jejunum loop.

### Western Blot Analysis of GLUT2 and GLUT5 in Membrane Preparations

16-hour fasted rats were anesthetized by pentobarbital and laparotomized for *in situ* experiments. Jejunal loops were isolated *in situ* and filled with a Krebs buffer without or with leptin. After 3 min of incubation, these loops were injected with 100 mM unlabelled galactose or 30 mM fructose. After a further 5 min incubation, the loops were removed, opened along the mesenteric border and the mucosa was scrapped off on ice. Total proteins and brush border membranes (BBM) were prepared in PBS containing protease and phosphatase inhibitors (Sigma Chemicals, St Louis Mo, USA) as previously described [9] and enrichment was estimated by determination of alkaline phosphatase activity (20-fold increase of activity in BBM). Protein concentration was quantified using the BCA protein assay Kit (Pierce, Rockford Il, USA) and used for western blot analysis. Solubilised proteins (50–100 µg) were resolved on SDS-PAGE in gels containing 8–10% acrylamide, transferred to nitrocellulose membranes and subjected to immunoblotting with rabbit polyclonal antibodies anti-GLUT2 (sc-9117) and anti-GLUT5 (sc-9117) [Santa Cruz Biotechnology, Inc., CA, USA). Immune complexes were detected by enhanced chemiluminescence (ECL; Perkin Elmer Boston, MA, USA). The intensity of the specific immunoreactive bands was quantified with Scion image (NIH, Scion Corporation, Bethesda, MD, USA). The results were expressed in relation to control, and the mean value of controls was arbitrarily set to 1 and used for calculations.

### Glucose Transporters in the Jejunum of AMPKα_2_^−/−^ Mice

AMPKα_2_^−/−^ mice were killed by cervical dislocation and the jejunum was removed for histological analysis by routine techniques. Sections (4 µm) were mounted on glass slides, stained with hematoxylin-eosin (H&E) and examined by light microscopy. Concerning Western blot analysis, proteins were extracted from jejunal mucosa scrapings as described above. For functional studies, a third jejunum fragment was used for SGLT-1 mediated glucose transport using the Ussing chamber as described in Figure S2.

### Acute Effects of Leptin on Jejunal GLUT2 and GLUT5 mRNA Levels

18-hour fasted mice were treated with vehicle or 3 ng/g murine leptin administered by gavage. Four hours later, blood was collected from the retro-orbital sinus with heparinized capillary Pasteur pipettes, centrifuged and the plasma was stored at −20°C. Mice were sacrificed, a fragment of their liver was removed and immediately frozen in liquid nitrogen. The jejunum was removed, rinsed and the mucosa was scrapped off and immediately frozen. All the collected tissues were stored at −80°C until use for extraction of total RNA for qRT-PCR analysis and for extraction of total protein for western blot analysis as described above, using the following antibodies: rabbit anti-fructose-1,6-bisphosphatase (anti-FBPase) polyclonal antibody (sc-66946), rabbit anti-FAS polyclonal antibody (sc-1023), goat anti-glucose-6-phosphatase (anti-G6Pase) polyclonal antibody (sc-33839) purchased from Santa Cruz (Santa Cruz, CA) and rabbit purified anti-ACC-1 monoclonal antibody and rabbit purified anti-phospho ACC-1 (Ser79) polyclonal antibody were purchased from Millipore (Millipore SAS, Molsheim, France).

### Chronic Effects of Leptin on GLUT2 and GLUT5 mRNA Levels in Mice Fed a High Fat Diet (HFD)

Experiments were performed on 6-week-old male C57BL/6J purchased from Janvier (Le-Genest-St-Isle, France) that were fed *ad libitum* a standard diet (SD: A04 biscuits; UAR, Villemoisson-sur-Orge, France) or a high-fat high-sucrose diet (HFD) purchased from SAFE, Augy, France. The HFD diet includes 36% fat, 35% carbohydrates mainly saccharose and 18% protein [51]. Mice fed on a SD or a HFD were treated every day with 3 ng/g leptin for 7 days by gavage. At day 8, blood was collected from non-fasted mice, the jejunum mucosa was scrapped off as described above and used for qRT-PCR and western blot analysis of transporters.

### RNA Preparation and Real-Time PCR Analysis (qRT-PCR)

Total RNA was extracted from the jejunal mucosa samples or liver fragments with the Trizol reagent (Qiagen). Quantitative RT-PCR was carried out on Light Cycler System 480 (Roche Diagnostics) as previously described. The oligonucleotides primers sequences of the mouse genes studied (Table 2) were designed with Oligo 4 software and synthesized by Eurogentec (France).

**Table 2:** Sequences of primers used for real time quantitative PCR.

Gene name	Accession number	Primer sequences	
Facilitative glucose transporter number 2 (GLUT2)	NM_031197	F5′-ctgctcttctgtccagaaagc-3′	R5′-tggtgacatcctcagttcctc-3′
Facilitative glucose/fructose transporter number 5 (GLUT5)	NM_019741	F5′-gaagacacactgagccgtgga-3′	R5′-cctttcttcagcagggaagtgtc-3′
Sodium/glucose co-transporter member 1 (SGLT1)	NM_019810	F 5′-gacatcccagaggactccaa-3′	R5′-accactgtcctccacaaagg-3′
Sterol regulatory element binding protein transcription factor 1c (SREBP-1c)	NM_011480	F5′-atcggcgcggaagctgtcgg-3′	R5′-actgtccttggttgttgatgagt-3′
Acetyl-CoA carboxylase 1 (ACC-1)	NM_133360	F5′-caatcttcctgcagcacagctcc-3′	R5-cccaaggagataccccatacatc-3′
Phosphoenolpyruvate carboxykinase (PEPCK)	NM_011044	F5′-gagtagcacagagaacag-3′	R5-tgactttgaagtggaaccca-3′
Ribosomal RNA (18S)	NM_111457	F5′-ccctgccctttgtacacacc-3′	R5′-gatccgagggcgctcacta-3′
Glucose-6-phosphatase (G6Pase)	NM_008061	F5′-cacaccaccttctctatcac-3′	R5-gttgcctaccagacacagc-3′
Fructokinase (FK)	NM_008439	F5′-tttgtgtccattccccaaat-3′	R5′-gtggactcccagttctgag-3′
Fasting-induced adipocyte factor (FIAF)	NM_020581	F5′-caggctaccaccctgttgat-3′	R5′-ctttgtccacaagacgcaga-3′

### Oral Fructose Tolerance Test

Three sets of experiments were conducted with the 16-hour fasted mice. In the first set of experiments, we determined whether leptin could increase fructose absorption in conscious animals. To this end, mice were given an oral load of 2 g fructose per kg of body weight added with 0.1 µCi radiolabelled [^14^C]-fructose without (control) or with 3 ng/g leptin. Before starting the oral fructose tolerance test (OFTT), blood samples were taken from the tail and blood glucose levels were determined (ACCU-CHEK; Roche Diagnostics, Meylan, France). The bleeds were further taken at 15, 30, 60, 90 and 120 min after administration. At 120 minutes, blood was collected, mice were sacrificed and segments of jejunum and the liver were removed. The radioactivity was counted and used to calculate the amount of fructose in these compartments. For jejunum and the liver, the data were expressed as moles of fructose per gram wet tissue.

The second set of experiments was designed to determine if the effects of leptin depend on the amount of ingested fructose. Mice were given an oral load of 1 g or 2 g unlabelled D-fructose per kg of body weight without (control) or with 3 ng/g leptin. Blood samples were taken and blood glucose levels were determined as mentioned above. These experiments were performed with 16 individual animals, each animal being its own control. The animals were allowed to recovery during 72 hours between two experiments.

The third set of experiments, fasted mice were divided into four groups and treated without or with fructose in the presence or the absence of 3 ng/g leptin. Fifteen minutes later, blood was drawn from the retro-orbital sinus with heparinised capillary Pasteur pipettes, centrifuged and the plasma was stored at −20°C until determination of insulin levels by RIA.

### Effects of Fructose on Luminal Leptin Contents from Stomach and the Intestine

18-hour fasted mice were treated by gavage with saline, fructose (1 g and 2 g/kg body weight), 6 g/kg galactose or with intraperitoneal injection of 10 ng/g CCK-8 as positive control. Fifteen minutes later, blood was drawn from the retro-orbital sinus, centrifuged and the plasma stored at −20°C until RIA of leptin. Mice were sacrificed, their stomachs and their small intestines (from duodenum to ileum) were removed and then flushed each with 1 ml of PBS buffer containing 500 UI/ml aprotinin; the luminal contents were collected on ice, centrifuged at 3,000 rpm for 10 min and the supernatants were used for a leptin determination by a RIA.

### Statistical Analysis

All values are expressed as means±SEM. One- or Two-way ANOVA followed by Tukey-Kramer multiple comparisons post-test was used to compare the groups. Statistical analysis was performed with Graph Pad Prism version 3.0 for Windows (Graph Pad Software, San Diego, CA, USA). Values of P<0.05 were considered statistically significant for all analyses.

## Supporting Information

Text S1 Luminal leptin increases GLUT2-mediated galactose transport and increases galactose levels in blood, in vivo in the rat. AMPKα_2_ KO mice had an increased expression and activity of SGLT1 in the jejunum.
(0.04 MB DOC)

Figure S1 Appearance of radiolabelled [14C]-galactose in blood after introduction into the jejunum. A solution of 100 mM galactose added with 0.1 µCi [14C]-galactose solution with saline or with 5 nM leptin alone or in association with 10 nM L39A/D40A/F41A leptin mutein (L39), was injected into jejunal loops from fasted rats fitted with carotid catheter as previously described [14]. Plasma galactose was significantly higher after treatment with luminal leptin than after treatment with vehicle. Results show incremental area under the curves (insert).*P<0.05 vs. saline, # vs. leptin.
(3.39 MB TIF)

Figure S2 Left panel: Western blot analysis of SGLT1 proteins in jejunum mucosa extracts from AMPKα_2_−/− and WT mice. Densitometric analysis of the blots was performed using NIH Image software and data are expressed as described above. Right panel: Effect of luminal leptin on SGLT1 transport activity in jejunum fragments from AMPKα_2_−/− and WT mice mounted in Ussing chamber. Vehicle or leptin was added in the mucosal bath 2 minutes before challenge with 10 mM glucose. Electrogenic chloride secretion in response to carbachol (100 µM) was used as a control. Values for Isc are expressed as mean±SEM of net increase in Isc (ΔIsc) in µA/cm2; n = 5 mice in each group. *P<0.05 and **P<0.01 vs. control.
(3.77 MB TIF)

Figure S3 Summary of the mechanisms involved in the luminal leptin regulation of GLUT5 and GLUT2 transporters. Luminal leptin operating through leptin receptors phosphorylates/activates ERK, AMPKα, and PKCβII leading to recruitment of more GLUT2/GLUT5 into the BBM and the subsequent increase in galactose and fructose transport across the jejunum into the blood. Thereafter, leptin replenishes the cytoplasmic pool of these transporters by increasing GLUT2 and GLUT5 mRNA levels.
(6.90 MB TIF)
