# Supplementary material for: Positive Regulatory Control Loop between Gut Leptin and Intestinal GLUT2/GLUT5 Transporters Links to Hepatic Metabolic Functions in Rodents
Source: PLoS One. 2009 Nov 30;4(11):e7935. doi: 10.1371/journal.pone.0007935 (PMC2780353; doi:10.1371/journal.pone.0007935)
Supplement: Text S1 — Luminal leptin increases GLUT2-mediated galactose transport and increases galactose levels in blood, in vivo in the rat. AMPKα2 KO mice had an increased expression and activity of SGLT1 in the jejunum. (0.04 MB DOC) [file pone.0007935.s001.doc]

**Luminal leptin increases GLUT2-mediated galactose transport and increases galactose levels in blood, *in vivo* in the rat.** We introduced into isolated jejunal loops *in situ*, a solution of 100 mM galactose containing radiolabelled [^14^C]-galactose and took blood samples from the carotid artery at various time-points as previously described [14]. We found a rapid and time-dependent increase of [^14^C]-galactose in the blood. This effect was further enhanced when 5 nM leptin was added directly into the jejunum (fig S1).

Interestingly, the addition of L39A/D40A/F41A mutein, a leptin-receptor antagonist, prevented the leptin stimulation of galactose appearance in blood. This indicates that luminal leptin stimulation of GLUT2-mediated galactose transport is leptin-receptor specific. Measurement of the area under curve AUC_0–30min_ revealed a significant 65% increase in [^14^C]-galactose in leptin-treated samples (P<0.01 *vs.* CTRL). ^14^C-galactose levels remained high at 120 min, with an AUC_0–120min_ of 30% (P<0.05 *vs.* CTRL) (fig S1, insert). With addition of 10 nM L39A/D40A/F41A mutein, this increase did not occur.

**AMPKα_2_ KO mice had an increased expression and activity of SGLT1 in the jejunum.** We took advantage of the AMPKα_2_-/- mice to analyse concomitantly with GLUT2/GLUT5, the expression and activity of SGLT1 transporter in the jejunum. As shown in fig S2, left panel, amounts of SGLT1 transporter proteins were increased in AMPKα_2_-/-, with levels two times higher than in WT mice. The transport activity of SGLT1 was determined in Ussing chamber (fig S2, right panel) as previously described. The addition of 10 mM glucose into the mucosal bath led to a rapid and significant rise in the short circuit current (*Isc)* which was 2.3 times higher in jejunum from AMPKα_2_-/- mice than in jejunum from WT mice (P<0.01). It can be noted that the mucosal leptin inhibition of SGLT1 transport activity (35%; P<0.05) consistent with previous finding [9] does not occur in jejunum from AMPKα_2_-/- mice. This suggests that the leptin inhibition of SGLT1 is dependent upon the α_2_ catalytic subunit of the AMPK.
