# Supplementary figures and images for: Positive Regulatory Control Loop between Gut Leptin and Intestinal GLUT2/GLUT5 Transporters Links to Hepatic Metabolic Functions in Rodents
Source: PLoS One. 2009 Nov 30;4(11):e7935. doi: 10.1371/journal.pone.0007935 (PMC2780353; doi:10.1371/journal.pone.0007935)

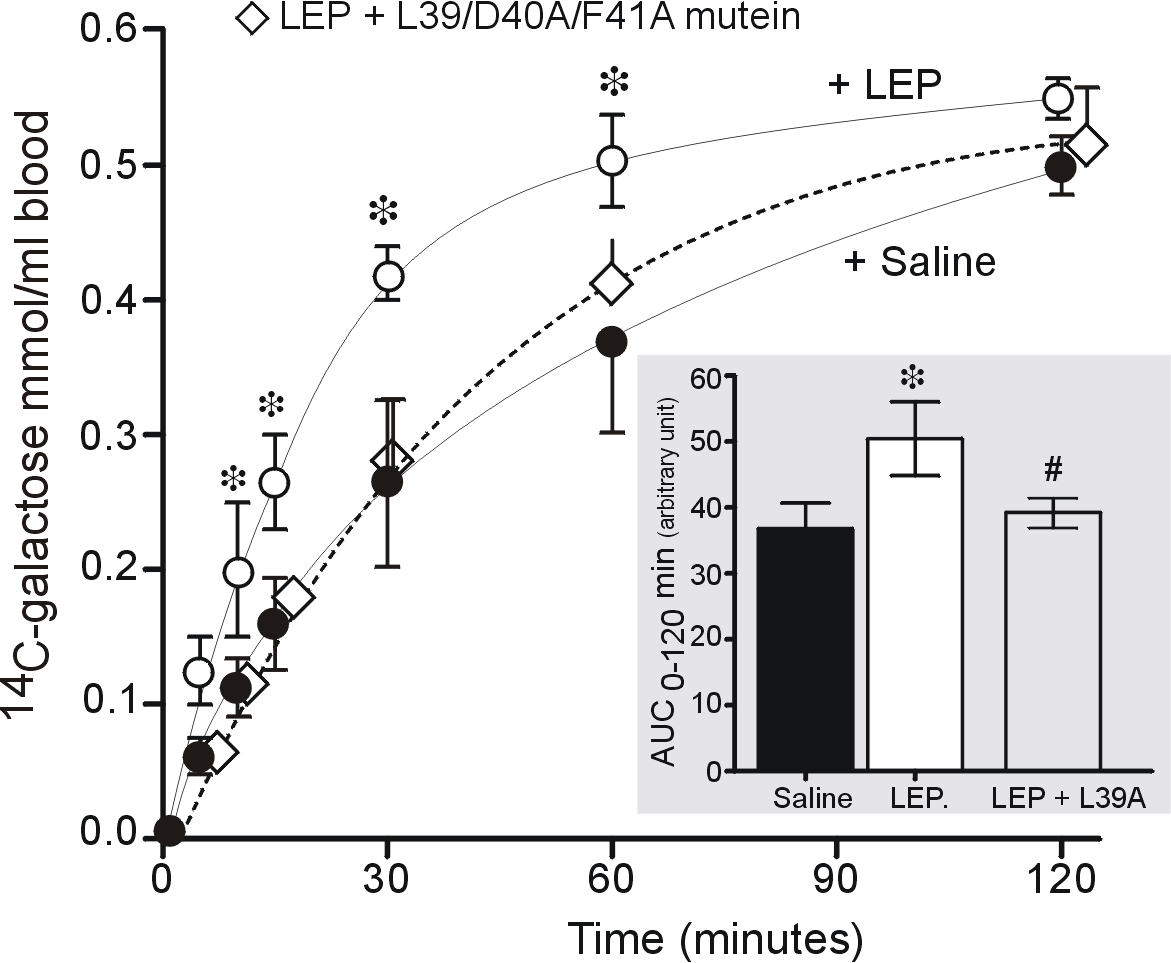

Supplement: Figure S1 — Appearance of radiolabelled [14C]-galactose in blood after introduction into the jejunum. A solution of 100 mM galactose added with 0.1 µCi [14C]-galactose solution with saline or with 5 nM leptin alone or in association with 10 nM L39A/D40A/F41A leptin mutein (L39), was injected into jejunal loops from fasted rats fitted with carotid catheter as previously described [14]. Plasma galactose was significantly higher after treatment with luminal leptin than after treatment with vehicle. Results show incremental area under the curves (insert).*P<0.05 vs. saline, # vs. leptin. (3.39 MB TIF) [file pone.0007935.s002.tif]

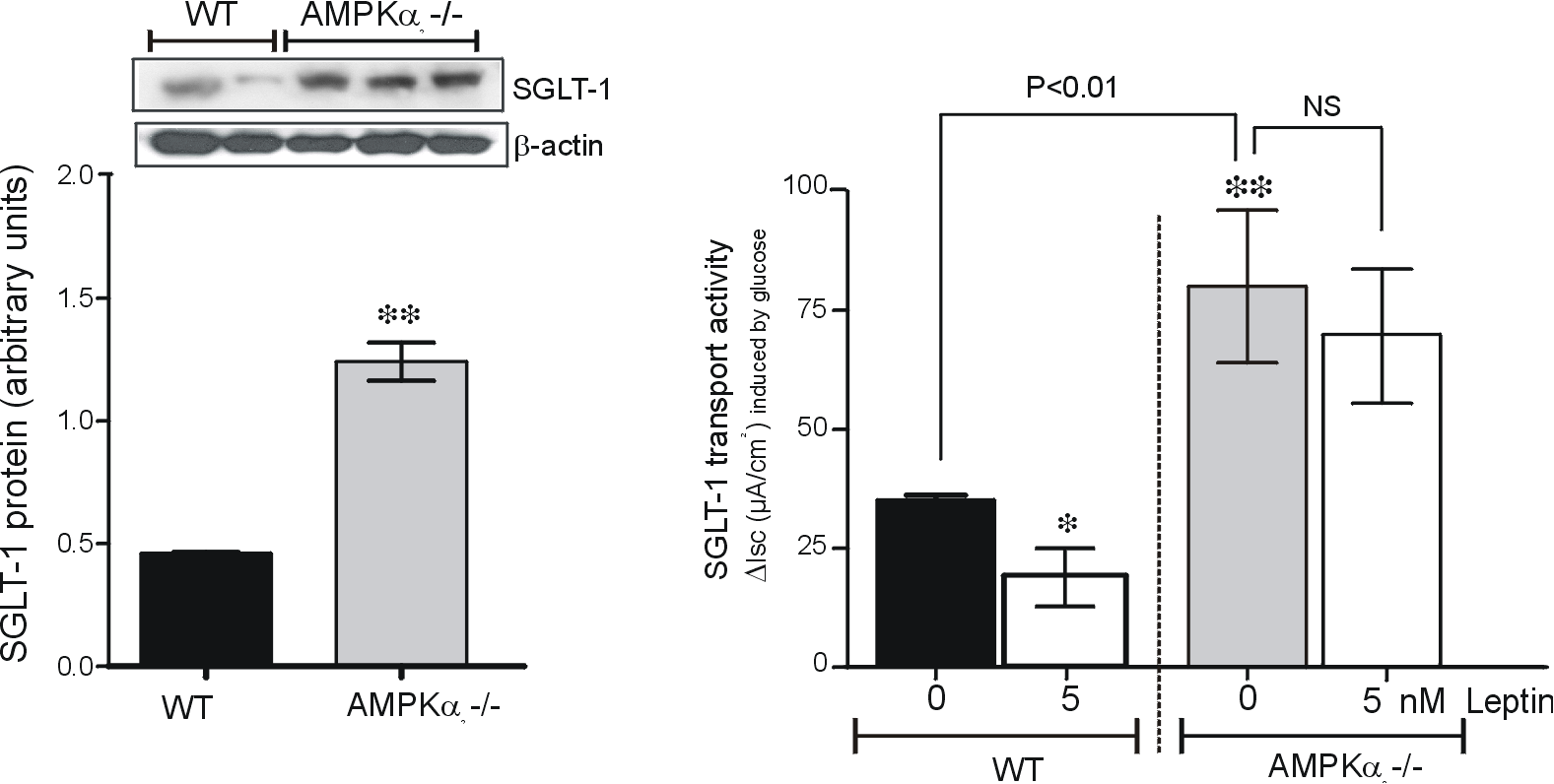

Supplement: Figure S2 — Left panel: Western blot analysis of SGLT1 proteins in jejunum mucosa extracts from AMPKα2−/− and WT mice. Densitometric analysis of the blots was performed using NIH Image software and data are expressed as described above. Right panel: Effect of luminal leptin on SGLT1 transport activity in jejunum fragments from AMPKα2−/− and WT mice mounted in Ussing chamber. Vehicle or leptin was added in the mucosal bath 2 minutes before challenge with 10 mM glucose. Electrogenic chloride secretion in response to carbachol (100 µM) was used as a control. Values for Isc are expressed as mean±SEM of net increase in Isc (ΔIsc) in µA/cm2; n = 5 mice in each group. *P<0.05 and **P<0.01 vs. control. (3.77 MB TIF) [file pone.0007935.s003.tif]

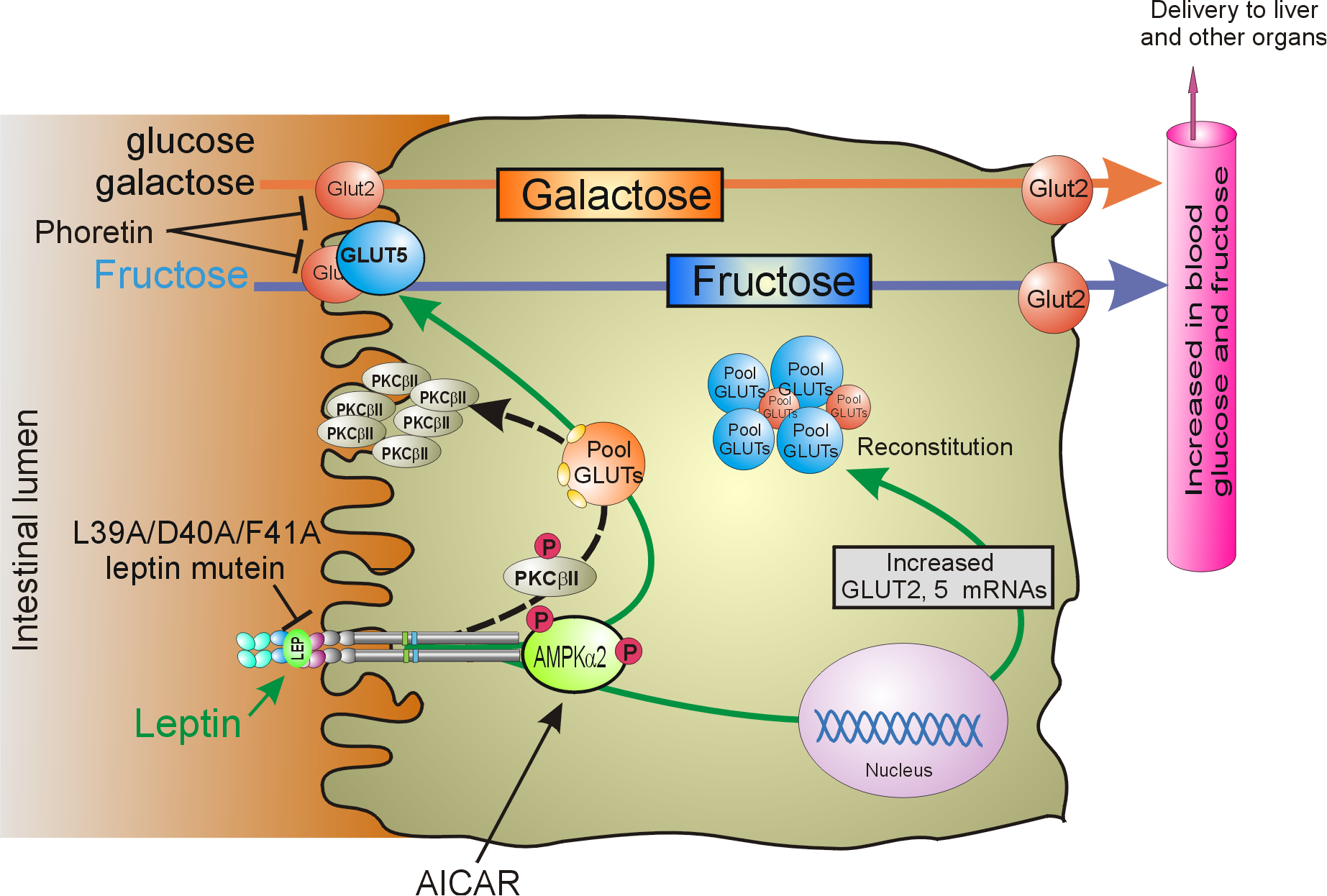

Supplement: Figure S3 — Summary of the mechanisms involved in the luminal leptin regulation of GLUT5 and GLUT2 transporters. Luminal leptin operating through leptin receptors phosphorylates/activates ERK, AMPKα, and PKCβII leading to recruitment of more GLUT2/GLUT5 into the BBM and the subsequent increase in galactose and fructose transport across the jejunum into the blood. Thereafter, leptin replenishes the cytoplasmic pool of these transporters by increasing GLUT2 and GLUT5 mRNA levels. (6.90 MB TIF) [file pone.0007935.s004.tif]
